# Statin-Induced Anti-3-Hydroxy-3-Methylglutaryl-Coenzyme A Reductase (Anti-HMGCR) Myopathy

**DOI:** 10.7759/cureus.34121

**Published:** 2023-01-23

**Authors:** Fatima Ghazal, Michelle Zur, Aaron Silver

**Affiliations:** 1 Internal Medicine, University of Connecticut Health, Farmington, USA; 2 Hospital Medicine, Hartford Hospital, Hartford, USA

**Keywords:** critical illness myopathy, drug-induced myopathy, immune-mediated necrotizing myopathy, statin safety, statin-associated muscle symptoms, statin-induced myopathy, anti hmgcr antibody, anti-hmgcr myopathy, transthyretin amyloidosis, statin-induced necrotizing autoimmune myopathy

## Abstract

Statins are known to pharmacologically target 3-hydroxy-3-methylglutaryl-coenzyme A reductase (HMGCR). Several subtypes of anti-HMGCR autoimmune myopathies have been reported as a result of statin use. Although these types vary widely, a severe and rare form of statin-induced myopathy is immune-mediated necrotizing myopathy (IMNM), resulting in severe muscle injury that does not respond to statin cessation and is associated with poor outcomes. Diagnosis is confirmed through biopsy confirming the necrosis of biopsy fibers, in addition to elevated anti-HMGCR serum levels. Management lacks proper guidelines, however, immunosuppressive therapy has been proposed as a possible intervention. The aim of this report is to increase providers’ knowledge of the presentation and possible treatment of statin-induced immune-mediated necrotizing myopathy.

## Introduction

Immune-mediated necrotizing myopathy (IMNM) is one of the three subtypes of autoimmune myopathies along with polymyositis and dermatomyositis [1]. It has been classified by the New European Neuromuscular Centre (ENMC) Criteria into three different sub-entities including anti-signal recognition particle (SRP) myopathy, anti-3-hydroxy-3-methylglutaryl-coenzyme A reductase (anti-HMGCR) myopathy and sero-negative IMNM [2]. Histological analysis of skeletal muscle differentiates IMNM from other subtypes of autoimmune myositis showing fiber necrosis and regeneration without inflammatory cells [3,4]. In patients with anti-SRP myopathy, the anti-SRP autoantibody levels directly correlate with disease severity and tend to have both heart and lung involvement and high resistance to steroid therapy [5].

The anti-HMGCR autoantibody subtype was discovered in 2010. Ninety percent of patients with this subtype are above the age of 50 and were on statin therapy [4]; however, a small number of patients with anti-HMGCR IMNM had no previous statin exposure [6]. This type of IMNM has acute-subacute onset defined as a disease course that develops within a few weeks to less than six months, respectively with creatine kinase (CK) level that is more than 30 times the upper limit and correlates with disease severity [7]. A proximal pattern of muscle weakness in the bilateral upper and lower extremities is noted that is usually milder than that in anti-SRP myopathy, with muscle weakness being more prominent in lower limbs than upper limbs in both subtypes [7]. Dysphagia is also observed, however, with less incidence when compared to the anti-SRP subtype [7,8]. However, statin cessation alone is not a sufficient treatment and patients often require immunosuppressive therapy [9].

We describe a case of an elderly male on many years of statin therapy who presented with significant lower extremity weakness and inability to walk despite being mobile at baseline and found to have anti-HMGCR-positive IMNM. This patient had significant debilitation, challenging diagnosis, and ultimately expired from his condition. Our goal is to enrich providers’ knowledge of the diagnosis and treatment of this condition and improve patient outcomes.

## Case presentation

An 85-year-old man with a medical history of atrial fibrillation, hypertension, and hyperlipidemia managed on atorvastatin 40 mg daily for four years, presented with new and progressively worsening bilateral lower extremity weakness for one month. Previously, he was in his usual state of health and used a cane with ambulation at baseline due to chronic history of bilateral knee osteoarthritis. He reported a fall two months prior to presentation, followed by worsening bilateral lower extremity weakness and dysphagia. There was no concern for saddle anesthesia, altered sensation, chest pain, or any urinary or fecal incontinence. Physical examination revealed bilateral upper extremities with motor strength of 5/5 distally and 4/5 proximally, and bilateral lower extremities with motor strength of 4/5 distally and 3/5 proximally. Cardiac, respiratory, and abdominal examinations were unremarkable, except for irregular rhythm. Vital signs were normal with blood pressure of 122/42 mmHg, heart rate of 72 bpm, respiratory rate of 18 breaths per minute, and temperature of 36.6 Celsius. Laboratories were significant for leukocytosis of 16.4k, creatinine of 0.7 mg/dl, CK of 2169 U/L, high sensitivity troponin of 1608 ng/L, erythrocyte sedimentation rate (ESR) 56 mm/h, and C-reactive protein (CRP) 2.90 mg/L. His ECG revealed atrial fibrillation with left axis deviation. Computed tomography (CT) of the spine done at an outside facility revealed severe degenerative changes at L5-S1 with new cortical irregularity and destruction of the superior endplate of L5; raising suspicion for osteomyelitis versus discitis. Magnetic resonance imaging (MRI) of the cervical, thoracic, and lumbar spine had no evidence of discitis or osteomyelitis, but noted diffuse symmetric edema and enhancement of the bilateral psoas muscles. The patient was admitted to medicine service and fluid resuscitation was initiated for elevated CK. Further investigations with speech evaluation revealed significant pharyngoesophageal dysphagia. Echocardiography showed severe concentric cardiac hypertrophy with an abnormal global longitudinal strain at -14.6% in a pattern of relative apical sparring suggestive of amyloid cardiomyopathy.

**Figure 1:**
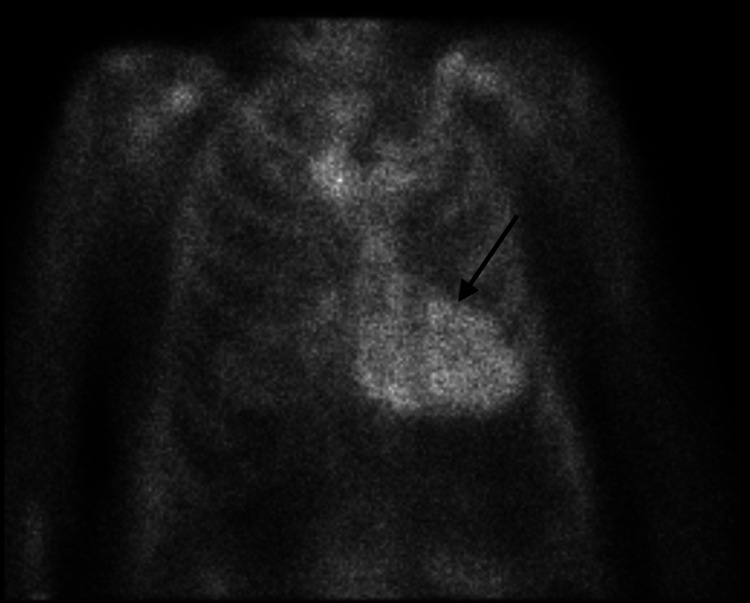
Pyrophosphate (PYP) scan strongly suggestive of transthyretin cardiac amyloidosis (ATTR-CM)

**Figure 2:**
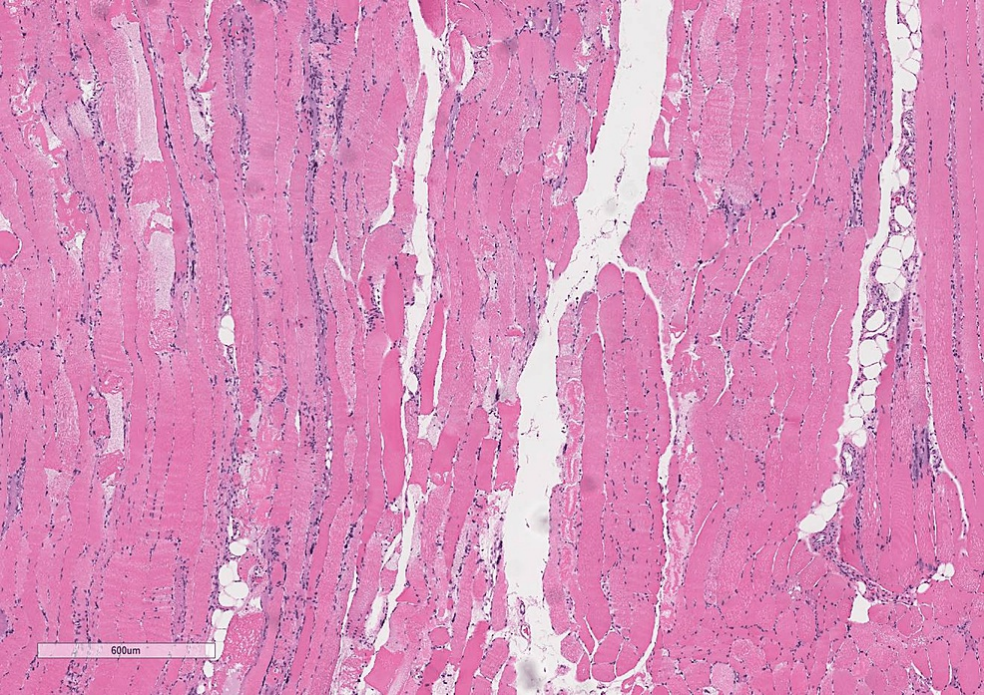
Hematoxylin and eosin stain of the left vastus lateralis muscle: Moderate variability in muscle fiber size with active myofiber necrosis and myophagocytosis with minimal inflammatory infiltration

**Figure 3:**
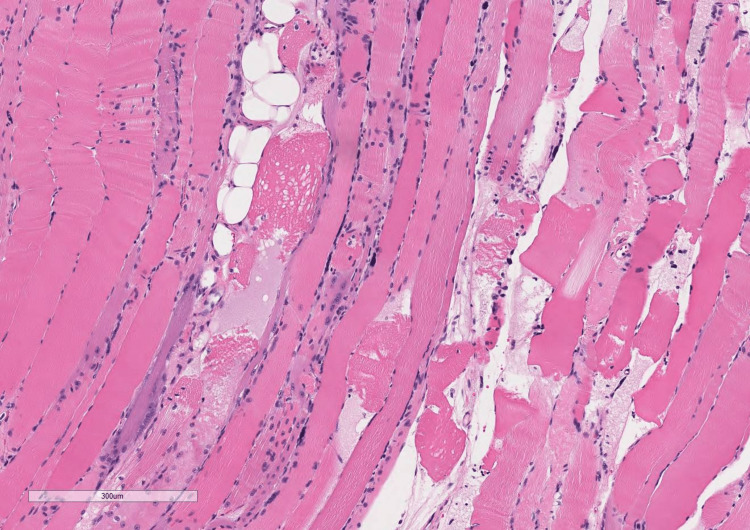
Hematoxylin and eosin stain of the left vastus lateralis muscle: Moderate variability in muscle fiber size with active myofiber necrosis and myophagocytosis with minimal inflammatory infiltration

Cardiology and heart failure services were consulted to assist further management. A pyrophosphate (PYP) scan was obtained and strongly suggested transthyretin cardiac amyloidosis (ATTR-CM) (Figure 1). Given concern for amyloidosis, further conducted rheumatological workup revealed normal serum protein electrophoresis with free kappa/lambda ratio of 1.21, elevated aldolase level of 28.5 U/L, thyroid-stimulating hormone (TSH) of 1.52 mIU/L, and normal immunoglobulin levels including IgG, IgA, and IgM with no abnormal bands noted on immunofixation. Ultimately, the patient was started on Solu-Medrol 250 mg IV for three days for possible immune-mediated process. Left vastus lateralis muscle and abdominal wall adipose tissue biopsies were obtained for the diagnosis of IMNM versus amyloidosis. Abdominal wall adipose tissue was negative for amyloid deposition. Left vastus lateralis muscle biopsy revealed moderate variability in muscle fiber size with active myofiber necrosis and myophagocytosis with minimal inflammatory infiltration on hematoxylin and eosin stain, significant for necrotizing myopathy (Figures 2, 3). Anti-HMGCR antibody was positive with a titer of 330, highly suggestive of statin-induced IMNM. The patient was discharged to a rehabilitation facility on prednisone 1mg/kg/day. He passed away prior to his rheumatology follow-up appointment at a skilled nursing facility.

## Discussion

Statin is a widely used medication for both primary and secondary prevention of coronary atherosclerotic cardiovascular disease. A well-known side effect is statin-associated muscle injury including myopathy, myositis, or rhabdomyolysis; all of which usually resolve with statin cessation. Alternatively, a much rarer side effect of statin therapy is immune-mediated necrotizing myopathy with positive anti-HMGCR antibodies. This type of injury does not respond to statin cessation alone and is often difficult to diagnose, thus causing increased morbidity and mortality.

The patient described in this report had both typical and atypical features of statin-mediated IMNM characterized by positive anti-HMGCR antibodies. Typical features included significant proximal muscle weakness, older age, chronic statin use, and elevated CK level in the 1000-2000 IU/L range. However, some of the more atypical features lead our team to explore other differentials and possibly delay treatment with steroids. There are rare reports of cardiomyopathy with IMNM; however, mimicking ATTR-CM is highly atypical [1]. Without cardiac muscle biopsy it is difficult to attribute the cardiac findings to IMNM rather than a separate finding of cardiac amyloidosis. However, the negative abdominal fat pad biopsy may stand to reason that these cardiac findings indeed are related to myopathy. Oropharyngeal dysphagia was reported as a differentiating feature between IMNM subtypes that is specific to anti-SRP autoantibodies [3]. Our patient with anti-HMGCR antibodies manifested this feature, which is a novel presentation. These features are important for clinicians to incorporate into their clinical acumen as delay in diagnosis and treatment can lead to lasting morbidity and even mortality.

Treatment for statin-induced IMNM is limited. The first step in treatment is statin cessation, although very minimal improvement is expected [9]. Corticosteroids including prednisone 1mg/kg/day or pulsed intravenous methylprednisolone of 250mg daily for three days remains the first line per the 224th ENMC International Workshop [2]. Other immunosuppressive therapies in combination with steroids such as rituximab have also been used but have little evidence behind them. One retrospective study from the University of Pittsburgh myositis database showed favorable outcomes with combination glucocorticoid and a variety of immunosuppressive therapies in anti-HMGCR positive patients [9]. More research is needed to guide appropriate treatment of IMNM. The aim of this report is to increase providers’ knowledge of the presentation and possible treatment of statin-induced immune-mediated necrotizing myopathy.

## Conclusions

Statin is a widely prescribed medication to reduce cholesterol levels that functions through inhibiting HMGCR. Muscle injury secondary to statin use is considered a commonly known adverse effect of the medication. Few reports describe IMNM as a complication of statin use that requires muscle biopsy for diagnosis confirmation. Anti-HMGCR-positive serum levels with elevated CK levels and a history of statin exposure should raise suspicion of this more severe form of muscle injury. IMNM is not responsive to discontinuation of the myotoxic medication and requires immunosuppression to aid with the resolution of symptoms. Nevertheless, more research needs to be done in order to identify the appropriate immunosuppressive regimen and aid in establishing guidelines. 
